# MicroRNA-30a regulates cell proliferation and tumor growth of colorectal cancer by targeting CD73

**DOI:** 10.1186/s12885-017-3291-8

**Published:** 2017-05-02

**Authors:** Minghao Xie, Huabo Qin, Qianxin Luo, Qunsheng Huang, Xiaosheng He, Zihuan Yang, Ping Lan, Lei Lian

**Affiliations:** 1 Department of Colorectal Surgery, The Sixth Affiliated Hospital, Sun Yat-sen University, 26 Yuancun Erheng Rd, Guangzhou, Guangdong 510655 People’s Republic of China; 2 Guangdong Provincial Key Laboratory of Colorectal and Pelvic Floor Diseases, The Sixth Affiliated Hospital, Sun Yat-sen University, Guangzhou, Guangdong 510655 People’s Republic of China; 3 Department of General Surgery, The Affiliated Hospital of Jiujiang University, Jiujiang, Jiangxi 332000 People’s Republic of China; 4 Guangdong Institute of Gastroenterology, The Sixth Affiliated Hospital, Sun Yat-sen University, Guangzhou, Guangdong 510655 People’s Republic of China

**Keywords:** MiR-30a, Colorectal cancer, Proliferation, Apoptosis, CD73

## Abstract

**Background:**

MicroRNAs are non-coding RNAs which regulate a variety of cellular functions in the development of tumors. Among the numerous microRNAs, microRNA-30a (miR-30a) is thought to play an important role in the processes of various human tumors. In this study, we aimed to explore the role of miR-30a in the process of colorectal cancer (CRC).

**Methods:**

The quantitative real-time PCR and western blot analysis were used to detect the expressions of miR-30a and CD73 in CRC cell lines and clinical tissues. The luciferase reporter assay was conducted to validate the association between miR-30a and CD73. The CCK-8, terminal deoxynucleotidyl transferase dUTP -biotin nick end labeling (TUNEL) assays and cell cycle flow cytometry were carried out to verify the biological functions of miR-30a in vitro. The nude mouse tumorigenicity experiment was used to clarify the biological role of miR-30a in vivo.

**Results:**

The expression of miR-30a was significantly reduced in tumor cells and tissues of CRC. The proliferation ability of CRC cells was suppressed and the apoptosis of cells was promoted when miR-30a is over-regulated, however, the biological effects would be inverse since the miR-30a is down-regulated. CD73 is thought to be a target binding gene of miR-30a because miR-30a can bind directly to the 3′-UTR of CD73 mRNA, subsequently reducing its expression. The proliferation suppression of the CRC cells mediated by miR-30a could be rescued after up-regulating the expression of CD73.

**Conclusions:**

MiR-30a plays an important role on regulating the cell proliferation and apoptosis, thus affecting the growth of the tumor in CRC. And it may participate in the disease process of CRC by regulating the expression of CD73.

**Electronic supplementary material:**

The online version of this article (doi:10.1186/s12885-017-3291-8) contains supplementary material, which is available to authorized users.

## Background

Colorectal cancer (CRC) is one of the common digestive malignancies whose incidence ranks the third in all tumors, and it is also a terrible tumor that can lead to death [1]. Although significant improvements have been achieved, the treatment of CRC is still a vital public health issue resulting in approximately 608,000 deaths annually [2]. There are still a lot of ambiguities in the molecular mechanism of CRC, further investigation is warranted to develop new effective therapeutic strategies. Nowadays, microRNAs (miRNAs) are thought to be crucial molecules for their role on regulating the expression of mRNA in different tumors [3, 4].

MicroRNAs are short non-coding RNAs and they can adjust the translation of their targeted mRNA through binding to the 3′ - untranslated regions (3′-UTRs) [5]. Accumulating evidence indicates that expression alterations of miRNAs are correlated with almost all human neoplasms, and that miRNAs may work as tumor suppressors as well as oncogenes [6, 7]. Friedman et al. [8] reported that over 60% of protein-coding genes are pairing to miRNAs in human. Furthermore, more than 50% of miRNA genes are found at the fragile sites and genomic regions involved in cancers, suggesting that miRNAs are intimately correlated with the pathogenesis of cancers, including cancer proliferation [9]. Recently, several studies indicated that miR-30a is down-regulated in multiple cancers [10–13] and that down-expression of miR-30a is correlated with a worse prognosis [13].

CD73 is a glycosylphosphatidylinositol-anchored membrane protein with a molecular weight of 70-kDa, which is also named for ecto-5′-nucleotidase [14, 15]. CD73 participates in the metabolism of extracellular ATP, and it can catalyze the hydrolysis of ATP/AMP into adenosine and phosphate together with CD39. Recently, CD73 was found to be elevated in a variety of tumor tissues, and associated with tumor angiogenesis, proliferation, as well as clinical characteristics and prognosis of cancer patients [16–18]. There are growing evidence indicating that CD73 might play a crucial part in cancer development [19].

Although there are several studies suggested that miR-30a and CD73 are respectively connected with CRC, no experiment is sufficiently definite the relationship between miR-30a and CD73 in CRC. We are the first to investigate the function of miR-30a in regulating CD73, thus affecting the growth of CRC. In this study, we hypothesized that miR-30a inhibits proliferation and accelerates apoptosis in CRC via suppression of CD73. We revealed that over-expression of miR-30a could inhibit proliferation and promote apoptosis of CRC cell both in vitro and in vivo, whereas down-expression of miR-30a showed reverse outcomes. Furthermore, we proved that CD73 may serve as a direct and functional target of miR-30a. We also identified that there is a negative correlation between the expression of miR-30a and CD73 in human CRC tissues. Our results indicated that CD73 is a target gene of miR-30a. Moreover, miR-30a may play a critical role in the occurrence and progression of CRC by regulating the expression of CD73.

## Methods

### Tumor specimens and cell culture

The tumor and adjacent control tissue specimens used in the study were prospectively collected from 27 consecutive CRC patients at the Sixth Affiliated Hospital of Sun Yat-sen University (Guangzhou, China) after surgical resection. The specimens were frozen in liquid nitrogen after resection. All samples collected and analyzed with informed consent obtained from the patients. The study protocol was approved by the Ethics Committee of the Sixth Affiliated Hospital of Sun Yat-sen University.

HEK293T cells and CRC cell lines, SW480, HCT116, LoVo, CaCo2, HT29, and RKO, were cultured in Dulbecco’s modified Eagle’s medium (DMEM) containing 10% fetal bovine serum (FBS). DLD1 and HCT8 cells were cultured in Roswell Park Memorial Institute-1640 medium supplemented with 10% FBS.

### RNA reversed transcription and quantitative real-time PCR (qRT-PCR) assays

Total RNA of clinical tissue specimens for polymerase chain reaction (PCR) were extracted with TRIzol reagent (Invitrogen) and RNAs were reversely transcribed by ReverTra Ace qPCR RT Kit (Toyobo Biochemicals) according to the instructions of reagents. Real-time PCR was carried out with GoTaq qPCR Master Mix (Promega) on the Applied Biosystems 7500 Sequence Detection system which uses the SYBR Green as the detection medium. All experiments are done at least three repetitions, and control reactions without cDNA templates were included. The U6 snRNA was chosen as the endogenous control in the detection of miRNA. The relative expression levels of each gene were calculated and normalized using the 2^-∆∆Ct^ method with reference to the expression of glyceraldehyde-3-phosphate dehydrogenase (GAPDH) or U6 snRNA. The specific primer sequences are shown in Table 1.

**Table 1:** Primer sequences of real-time PCR

Gene		Sequence (5′ - 3′)
CD73	Forward Primer	ATTGCAAAGTGGTTCAAAGTCA
	Reverse Primer	ACACTTGGCCAGTAAAATAGGG
GAPDH	Forward Primer	GAGTCAACGGATTTGGTCGT
	Reverse Primer	GACAAGCTTCCCGTTCTCAG
miR-30a	Forward Primer	GCGTGTAAACATCCTCGAC
	Reverse Primer	GTGCAGGGTCCGAGGT
U6	Forward Primer	CTCGCTTCGGCAGCACA
	Reverse Primer	AACGCTTCACGAATTTGCGT

### Western blot analysis

Standard western blot was performed to detect the expression level of proteins. Cells were lysed with radio-immunoprecipitation assay lysis buffer. We used the sodium dodecyl sulfate-polyacrylamide gel electrophoresis to separate the proteins, and then transferred these proteins to the polyvinylidene difluoride membranes (Millipore). After blocking with 5% skim milk (BD Biosciences), the membrane was incubated with mouse anti-CD73 (1:4000 dilution) and mouse anti-GAPDH (1:3000 dilution) antibodies.

### Plasmids, virus production and transduction

The pLV-puro lentivirus vector was chosen as a genetic vector, and the miR-30a precursor was cloned into its restriction enzyme cutting site. MiR-30a sponge which contains 6 tandem “bulged” miR-30a binding motifs was designed and cloned into the pLV-puro vector for the following experiments. The open reading frame (ORF) region of human CD73 was reconstructed with pMSCV-puro retroviral vector by cloning into the *EcoR-1/Bgl-2* sites. Using the kit of Lipofectamine 2000 reagent (Invitrogen), the plasmids were transfected into the target cells. As previously described, the cells that stably express miR-30a or miR-30a sponge (sequence: 5′-CTTCCAGTCACGATGTTTACACCGGCTTCCAGTCACGATGTTTACACCGGCTTCCAGTCACGATGTTTACACCGGCTTCCAGTCACGATGTTTACACCGGCTTCCAGTCACGATGTTTACACCGGCTTCCAGTCACGATGTTTACA-3′) were obtained though retroviral infection using the HEK293T cells [20].

### Luciferase reporter assay

The 3′-UTR region of human CD73 gene was cloned into the pGL3 luciferase reporter plasmid (Promega) at the sites of *Bgl-2/Xho-1*. Three thousand cells were seeded into the 48-well plates and then cultured for 24 h, and each group of cells had three replicates. Using the Lipofectamine 2000 reagent (Invitrogen), the Luciferase reporter plasmids (100 ng) together with pRL-TK renilla plasmids (1 ng) were transfected into the target cells. Twenty-four hours after transfection, the reporter activity was tested by using a Dual Luciferase Reporter Assay Kit (Promega).

### Cell proliferation, apoptosis, and cycle analysis

When the stable expression cells were successfully constructed, cell proliferation was detected at 24, 48, 72, and 96 h via Cell Counting Kit-8 (CCK8) based on manufacturer’s instructions. Briefly, a number of 1 × 10^4^ cells per well with a final volume of 100 μl were evenly inoculated into the 96-well plates. Subsequently, 10 μl CCK-8 solution was added to each well at the particular time. After incubation at 37 °C for 30 min, the absorbance at 450 nm were measured with a microplate reader. TUNEL assay was performed for the purpose of detecting the apoptotic cell death using DeadEnd™ Fluorometric TUNEL System (Promega). The Hoechst 33,258 (Invitrogen) was used to label the cell nucleus. For cell cycle assay, the cells were collected and fixed in ethanol with a concentration of 70%, then placed in a 4 °C refrigerator overnight. Cell cycles were analyzed by using the flow cytometry (Beckman Coulter) after stained with propidium iodide (Biolegend) solution at a terminal concentration of 50 μg/ml containing 50 μg/ml RNase A.

### Xenograft model in nude mice

Before animal experiments, we had submitted the animal experiment ethical application and obtained approval from the Institutional Animal Care and Use Committee of the Sun Yat-sen University. All operations were carried out in accordance with established rules and regulations. Ten million of tumor cells per mouse, including SW480-non-regulated control (NC), SW480-miR-30a, and SW480-miR-30a sponge were injected into the dorsal skin of 4–6 week-old BALB/c nu/nu mice (purchased from Experimental Animal Center of Sun Yat-sen University, six mice per group). All mice were housed and maintained under specific pathogen-free conditions. At the end of the experiment, the tumors were taken out and their weight were recorded after the mice were sacrificed.

### Statistical analysis

The data were analyzed by using the GraphPad Prism V software. *P* values were calculated with the statistical method of two-sided Student’s *t*-test. Since *P* values <0.05, the result was considered statistically significant. When *P* values <0.01, the result was considered highly significant.

## Results

### MiR-30a regulates cell proliferation and apoptosis in CRC cells

**Fig. 1:**
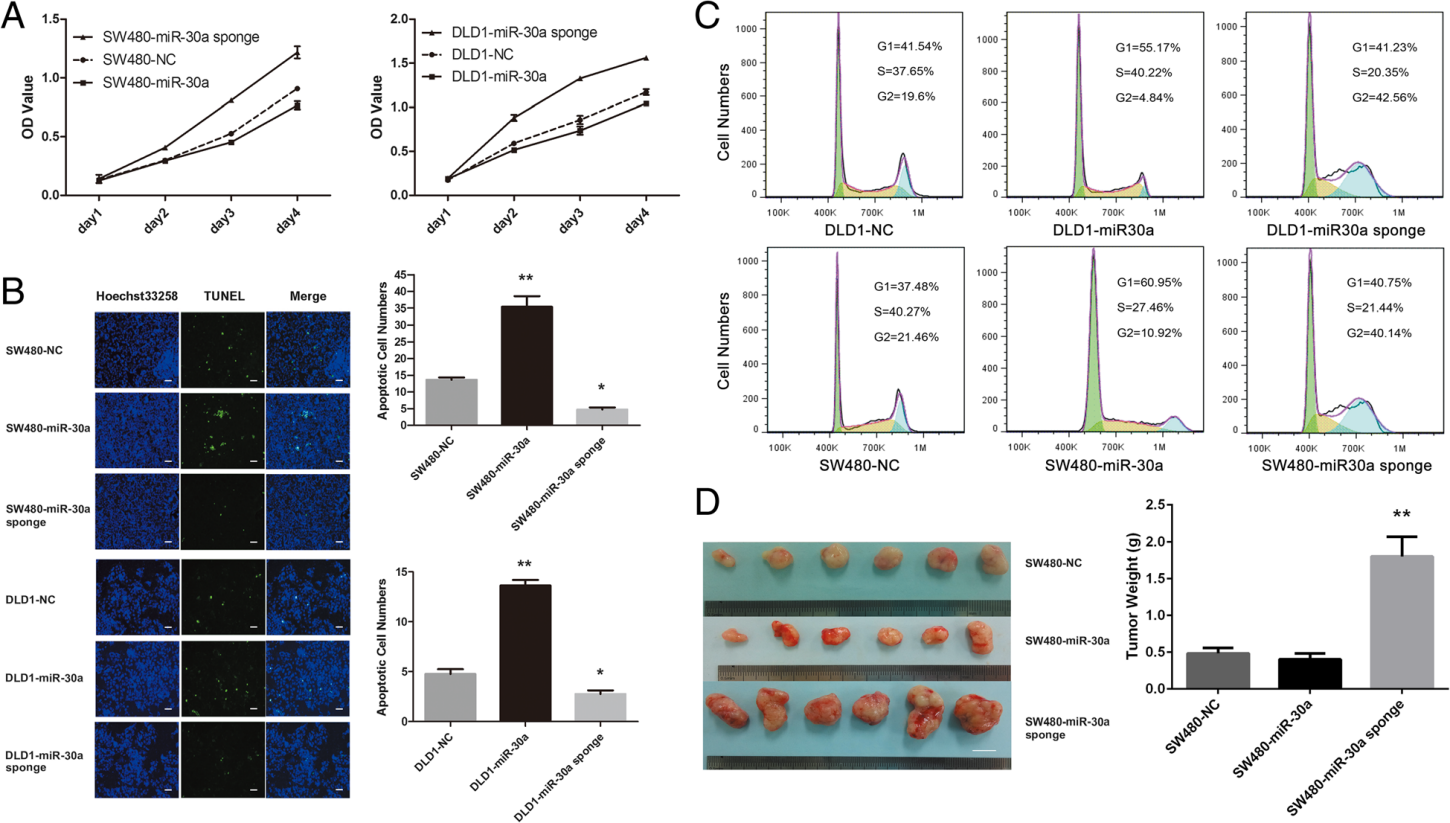
MiR-30a regulated CRC proliferation and apoptosis both in vitro and in vivo. **a** CCK-8 assays of SW480 (*left*) and DLD1 (*right*) cells with regulated expression of miR-30a﻿﻿. **b** Detection of apoptosis by TUNEL assays in different miR-30a expression CRC cells. *Blue*, Hoechst-stained nuclei; *green*, TUNEL-positive nuclei. Scale bar = 50 μm. **c** Over-expression of miR-30a in CRC cells blocked G1/S transition. The down-expression of miR-30a cells were activated in G2 phase of the cell cycle. **d** SW480-NC, SW480-miR-30a, and SW480-miR30a sponge cells were injected into the flanks of nude mice (*n* = 6). Tumor weights were recorded and assessed. Scale bar = 1 cm. *P < 0.05, **P < 0.01 compared with NC group. The CCK-8 assays were measured in five replicate values for each independent experiment. The TUNEL assays were calculating the numbers of apoptotic cells in one field, and we chose eight fields to calculate for each sample

The different expression levels of miR-30a and CD73 were firstly screened in 8 strain cell lines of CRC (SW480, HCT116, LoVo, CaCo2, HT29, RKO, DLD1 and HCT8) by qRT-PCR and western blot analysis (Additional file 1: Figure S1). To investigate whether miR-30a can affect CRC cell proliferation and survival, we stably over and down expressed miR-30a in SW480 and DLD1 cells. These cells were then used to determine their characters of proliferation and apoptosis. As shown in Fig. 1a, over-expression of miR-30a could significantly inhibit the proliferation ability of SW480 and DLD1 cells in CCK-8 assays, while down-expression of miR-30a displayed an opposite effect. In TUNEL assays (Fig. 1b), over-expression of miR-30a showed that it can significantly accelerate the apoptosis of CRC cells, and down-expression of miR-30a showed inverse results. Furthermore, we found that over-expression of miR-30a caused a G1 arrest and down-expression of miR-30a caused a G2 arrest by cell cycle analysis (Fig. 1c). These results demonstrate that miR-30a can suppress the proliferation and survival of CRC cells in vitro. To further investigate whether miR-30a shows the same effect in vivo, we injected SW480 cells with different expression of miR-30a (over, down and non-regulated control) into nude mice by subcutaneous injections. There were significantly differences in the mean weights of xenograft tumors between miR-30a down-expression and non-regulated control groups (Fig. 1d). On the whole, the above results indicate that miR-30a plays an important role in regulating the proliferation and apoptosis of CRC cells both in vitro and in vivo.

### CD73 is a direct target of miR-30a

**Fig. 2:**
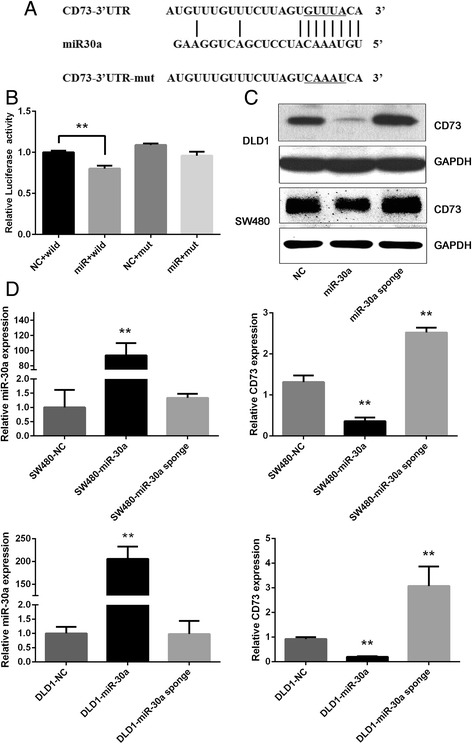
CD73 was a direct target of miR-30a. **a** Predicted miR-30a target sequences in the 3′-UTR of CD73 and its mutant containing altered nucleotides in the 3′-UTR. **b** The miR-30a target sequence from CD73 was cloned into the 3′-UTR of a luciferase reporter gene. Seed site mutagenesis was used to control for binding specificity. Luciferase activity was determined by Dual-Luciferase Reporter Assay System. **c** CD73 protein expression levels in CRC cells infected with miR-30a precursor or miR-30a sponge were determined by western blotting. **d** CD73 mRNA expression levels in CRC cells infected with miR-30a precursor or miR-30a sponge were determined by qRT-PCR. Error bars represent mean ± SD from three independent experiments. *P < 0.05, **P < 0.01 compared with the NC group. The luciferase reporter assay data were measured in triplicates for each independent transfection experiment

In order to determine the mechanism of miR-30a in regulating the proliferation and apoptosis of CRC cells, we next used several target prediction programs, TargetScan, miRWalk and PicTar, to explore the potential target gene of miR-30a. Results of analysis revealed that the 3′-UTR of CD73 mRNA has two complementary sites for miR-30a targeted binding (Additional file 2: Figure S2). To verify this prediction, human CD73 3′-UTR fragment with the wild-type or mutant miR-30a-binding site was inserted to the downstream of the open reading frame of luciferase. Dual-Luciferase Reporter Assay System (Promega) was used to detect the relative activity of luciferase. The luciferase reporter assay showed that only one of the two sites is the miR-30a binding site (Fig. 2a). As shown in Fig. 2b, the relative activity of luciferase in the reporter containing a wild-type CD73 3′-UTR was markedly decreased upon miR-30a co-transfection, whereas the reporter containing the mutant binding site was unaffected in the luciferase activity. Furthermore, the results of qRT-PCR and western blot analysis suggested that miR-30a has a negative effect in regulating the expression levels of CD73 mRNA and protein (Fig. 2c and d). As shown by these results, CD73 is a direct target gene of miR-30a in CRC cells.

### MiR-30a and CD73 expression levels in CRC tissue

**Fig. 3:**
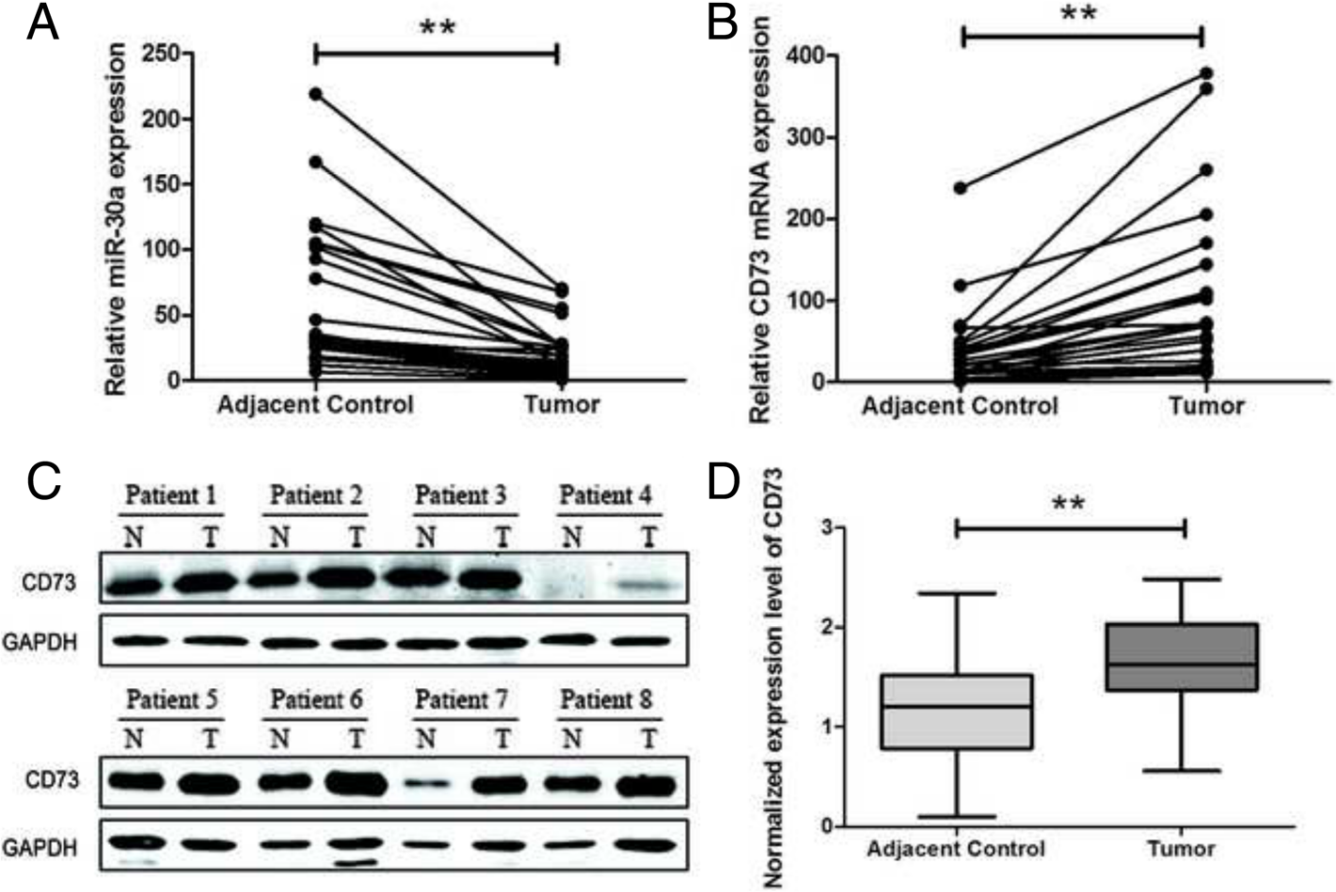
The inverse correlation between the expression levels of miR-30a and CD73 in 27 pairs of clinical specimens. qRT-PCR analyses of miR-30a (**a**) and CD73 (**b**) expression in CRC and corresponding adjacent control tissues. **c** CD73 protein expression levels in CRC tissues were determined by western blot (results of 8 patients were shown). **d** Densitometry analysis of western blot data normalized with GAPDH in all specimens (**P < 0.01)

To verify the relative expression of miR-30a and CD73, qRT-PCR and western blot was performed on 27 pairs of clinical specimens. At the mRNA level, tumor tissues showed lower expression levels of miR-30a and higher expression levels of CD73 than the corresponding adjacent control tissues, indicating a potential correlation between miR-30a and CD73 in CRC (Fig. 3a and b). At the protein level, western blot analyses showed similar results (Fig. 3c and d, Additional files 3 and 4: Figures S3 and S4). The results showed potential inverse correlations between the levels of miR-30a and CD73.

### CD73 is involved in miR-30a inhibited proliferation and survival of CRC cells

**Fig. 4:**
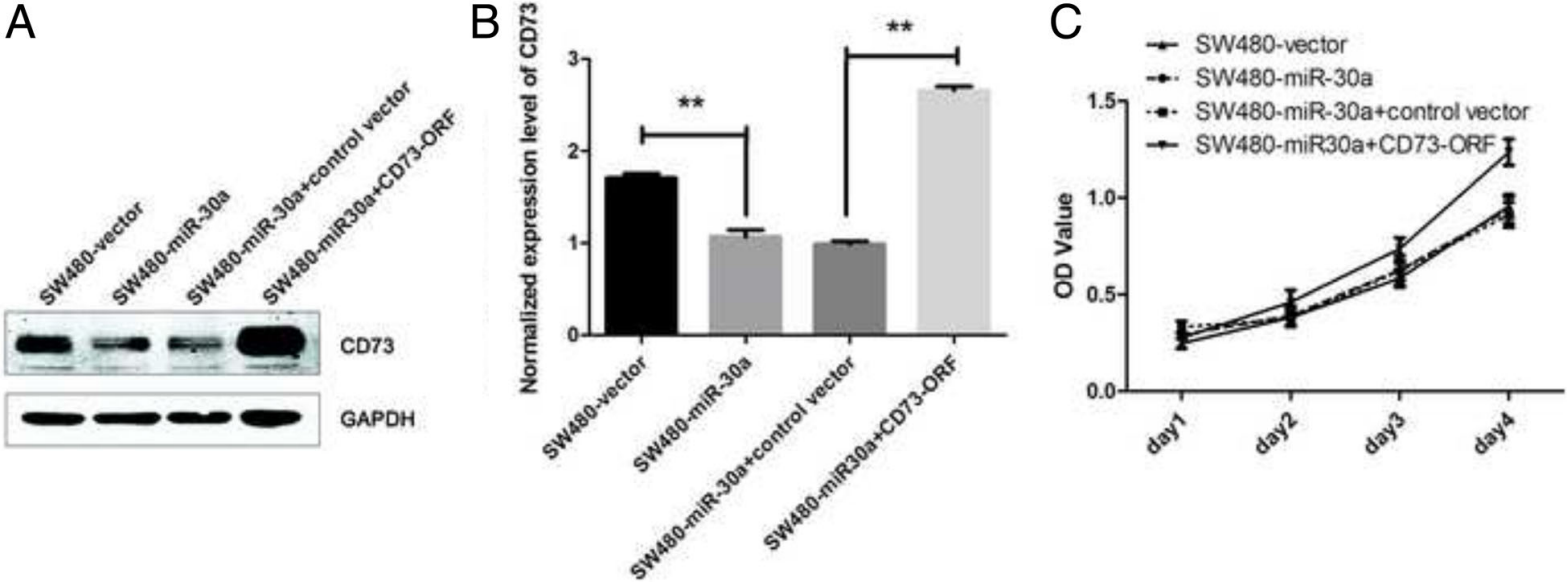
Over-expression of CD73-ORF rescues the ability of proliferation of the miR-30a over-expression CRC cells. **a** Western blot analyses of CD73 protein expression in SW480-vector cells, SW480-miR-30a cells, SW480-miR-30a cells transfected with control vector or CD73-ORF vector from three independent experiments. **b** Densitometry analysis of western blot data normalized with GAPDH (mean ± SD; *n* = 3; **P < 0.01). **c** CCK-8 assays of the cells

To further determine whether miR-30a regulates the proliferation and survival of CRC cells through CD73, we transfected miR-30a over-expression SW480 cells with CD73-ORF fragment (without 3′-UTR). The western blot analysis and qRT-PCR were used to verify the result of transfection (Fig. 4a and b). As shown in Fig. 4c, The CCK-8 assays indicated that ectopically expressing CD73 significantly promoted the proliferation of miR-30a over-expression SW480 cells. As these results shown, re-expression of CD73 can reverse the effect of miR-30a over-expression. CD73 is involved in miR-30a for inhibiting the proliferation of CRC cells.

## Discussion

In the present study, our data provide evidence that exogenously expressing miR-30a can significantly down-regulate the expression of CD73 mRNA and protein in CRC cells. Furthermore, we found that over-expression of miR-30a, which is frequently down-regulated in CRC, suppresses proliferation and promotes apoptosis of CRC cells through down-regulating the expression of CD73 in vitro. Xenograft tumor assays showed that it could significantly promote the growth of CRC when down-regulated the expression of miR-30a in vivo. On the other hand, reverse results were confirmed by inhibiting the expression of miR-30a.

It has been proven that miR-30a is one of important tumor-suppressor factors in various human cancers. The level of miR-30a is significantly decreased in multiple human tumors [21, 22]. Ouzounova et al. [22] showed that the expression of miR-30a was reduced in breast cancer via comprehensively analyzing the miR-30 family targets. In the present study, we revealed that miR-30a is also significantly reduced in CRC cell lines. This finding was confirmed by measuring the expression level of miR-30a in 27 clinical CRC specimens and their corresponding adjacent normal tissues using the method of qRT-PCR. Moreover, our results of cell cycle assays showed that the expression of miR-30a has a close association with the cell cycle of CRC cells. Over-expression of miR-30a blocked G1/S transition, while down-expression of miR-30a accelerated G2/M transition of CRC cells. All the above results demonstrated that miR-30a is critical in disease progression of CRC.

Several oncogenes have been identified as miR-30a targeted genes [10, 12, 13]. Boufraqech et al. [10] demonstrated that miR-30a decreases the expression level of lysyl oxidase in human anaplastic thyroid cancer. Zhang et al. [12] reported that miR-30a could suppress the growth of colon cancer cell by inhibiting the expression of insulin receptor substrate 2. However, the specific function of miR-30a in CRC is still largely unknown because of the lack of information on the target genes. We identified that CD73 was one of the direct target genes of miR-30a in CRC cells by luciferase reporter assay. Exogenously expressing miR-30a could significantly decrease the expression of CD73 mRNA and protein in CRC cells. In addition, our results indicated that miR-30a down-regulated the endogenous CD73 in CRC tissues as well.

It has been reported that CD73 is over-expressed in different tumors. In digestive system, some studies reported that over-expression of CD73, as a poor marker of clinical outcomes, was closely related with tumor differentiation, invasion and metastasis [15, 23]. Recently, CD73-adenosinergic metabolic pathway has been described as an vital immunosuppressive pathway involved in tumor progression [24, 25]. Stagg et al. [26] reported that CD73 deficiency inhibited the growth of prostate tumor and increased the amount of CD8^+^ T cells for infiltration. Accumulation of adenosine in the tumor microenvironment was found when there are over-expressing CD73, which considered as a new mechanism for immune escape of tumor [27, 28]. Tissue hypoxia and soluble factors in the tumor microenvironment were confirmed as the promoters of CD73-adenosinergic pathway [29].

However, at present, the specific miRNA targeting CD73 is remain unknown in CRC. Our data first showed that miR-30a may directly bind to the 3′-UTR of CD73 to regulate the proliferation of CRC cells both in vitro and in vivo. We designed different experiments in order to confirm the specific role of CD73 companied with miR-30a in mediating the functions associated with cell proliferation and tumor growth of CRC. Using lentivirus transfection to regulate the miR-30a expression, we showed that miR-30a and CD73 may have an important influence on both proliferation and apoptosis of CRC cells. Over-expression of CD73 can reverse the results of miR-30a up-regulation to enhance the proliferation of CRC cells. Furthermore, using xenograft tumor assays, we showed that down-expression of miR-30a not only suppressed the expression of CD73, but also significantly promoted the growth of xenograft tumor.

As a key immunosuppressive factor in tumor microenvironment, CD73 plays an important role in tumor growth. Anti-CD73 therapy becomes a potential treatment for various human cancers. In this regard, there are accumulating studies suggest that CD73 targeted therapy may be a novel method to effectively control the growth of tumor. Stagg et al. [17] reported that targeted therapy against CD73 using the anti-CD73 monoclonal antibody could suppress tumor growth and metastasis of breast cancer. In addition, Wang et al. [30] showed that CD73 selective inhibitor suppressed the growth of tumor and could effectively restore efficacy of adoptive T cell treatment in model mice of ovarian tumor as well as anti-CD73 monoclonal antibody. Meanwhile, miRNAs have been demonstrated to participate in cancer progression, and to affect therapeutic response and patient overall survival, thereby developing and exploiting miRNA-based therapeutics became endeavored fields of biomedical sciences [4]. In our present investigation, we found that miR-30a can suppress cell proliferation as well as tumor growth of CRC by regulating the expression of CD73. Therefore, miR-30a can be regarded as potential target for CRC therapy.

There are several limitations in our study. Firstly, we injected different miR-30a expression (over, down and non-regulated control) SW480 cells into mice by subcutaneous injections. The result showed that the mean weights of xenograft tumors between miR-30a down-expression and non-regulated control groups was significantly different. While there were not significantly different between miR-30a over-expression and non-regulated control groups. We think this result may because the basal expression of miR-30a is already very high in SW480 cells, and then over-expression of the gene may have little influence on its function. Secondly, by using target prediction programs, we predicted that the 3′-UTR of CD73 mRNA includes two complementary binding sites for the seed region of miR-30a. However, we only verified one of the two sites, position 1442–1449 of CD73–3’UTR, in which miR-30a can bind to the 3′-UTR of CD73 mRNA. The other one site, position 328–355 of CD73–3’UTR, which did not show a directly target (Additional file 5: Figure S5). Thirdly, we could not analyze the clinical outcomes because of limited samples and lack of clinical data. Nevertheless, the definite effect of miR-30a in regulation of CD73-adenosinergic pathway in CRC is unclear. The functions of miR-30a and CD73 in the complex signal path network of cell proliferation and apoptosis should be further explored. Studies based on large-scale samples are warranted to investigate the relevance of miR-30a expression levels to the prognosis and clinicopathological features of CRC patients.

## Conclusions

In conclusion, the data of this work provide new viewpoints about the role of miR-30a in human CRC. Our results firstly showed that miR-30a is down-regulated in CRC. And it inhibits cell proliferation and tumor growth in CRC by targeting CD73. Therefore, miR-30a may participate in the occurrence and development of CRC by regulating the expression of CD73.

## Additional files

Additional file 1: Figure S1. A. miR-30a expression assessed by Real-time PCR in eight CRC cell lines. B. CD73 expression assessed by western blot in eight CRC cell lines. (TIFF 1311 kb)

Additional file 2: Figure S2. CD73 sequence analysis indicated that putative miR-30a-binding sites were at 238–335 and 1442–1449 sequences of the CD73 3′-UTR. (TIFF 140 kb)

Additional file 3: Figure S3. The original results of western blot for the colorectal cancer tissues. (JPEG 153 kb)

Additional file 4: Figure S4. The results of western blot for the new collected colorectal cancer tissues. (TIFF 2972 kb)

Additional file 5: Figure S5. A. Wild-type (WT) and mutant (Mut) of putative miR-30a targeting sequences in CD73 mRNA. Mutant sequences were shown in underline. B. The miR-30a target sequence from CD73 was cloned into the 3′-UTR of a luciferase reporter gene. Seed site mutagenesis was used to control for binding specificity. Luciferase activity was determined by Dual-Luciferase Reporter Assay System. Error bars represent mean ± SD from three independent experiments. *P < 0.05, **P < 0.01 compared with the NC group. (TIFF 568 kb)

Additional file 6: Figure S6. The scan of informed consent for preservation of the tissue specimens in Chinese. (PDF 816 kb)

## Abbreviations

3′-UTR: 3′ - untranslated region
CCK-8: Cell counting kit-8
CRC: Colorectal cancer
DMEM: Dulbecco’s modified Eagle’s medium
FBS: Fetal bovine serum
GAPDH: Glyceraldehyde-3-phosphate dehydrogenase
miR, miRNA: microRNA
NC: Non-regulated control
ORF: Open reading frame
PCR: Polymerase chain reaction
TUNEL: Terminal deoxynucleotidyl transferase dUTP nick end labeling
