# Supplementary figures and images for: MicroRNA-30a regulates cell proliferation and tumor growth of colorectal cancer by targeting CD73
Source: BMC Cancer. 2017 May 2;17:305. doi: 10.1186/s12885-017-3291-8 (PMC5414330; doi:10.1186/s12885-017-3291-8)

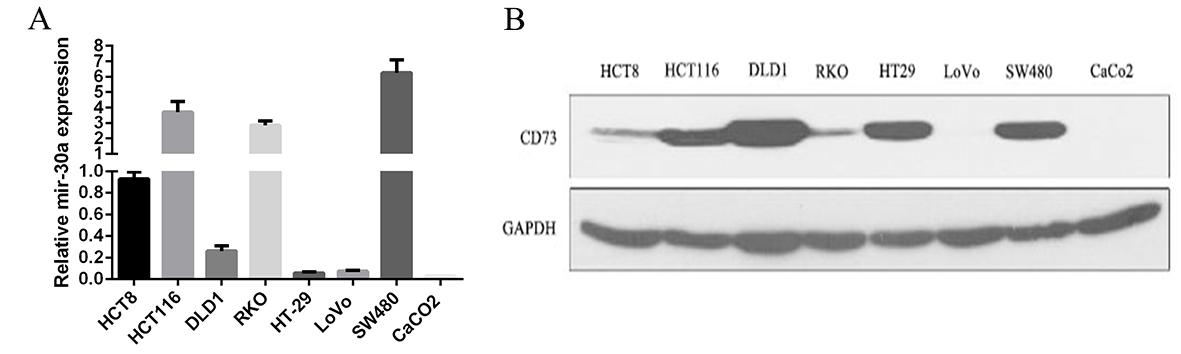

Supplement: Supplementary file 1 — A. miR-30a expression assessed by Real-time PCR in eight CRC cell lines. B. CD73 expression assessed by western blot in eight CRC cell lines. (TIFF 1311 kb) [file 12885_2017_3291_MOESM1_ESM.tif]

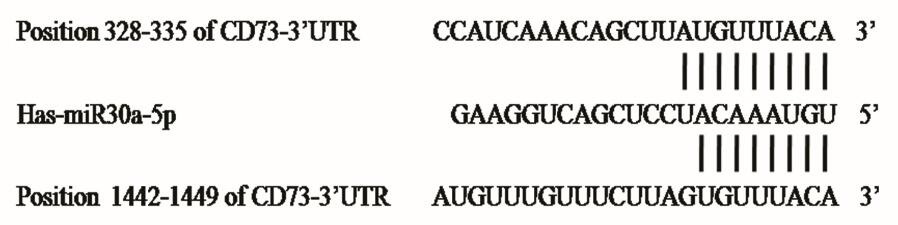

Supplement: Supplementary file 2 — CD73 sequence analysis indicated that putative miR-30a-binding sites were at 238–335 and 1442–1449 sequences of the CD73 3′-UTR. (TIFF 140 kb) [file 12885_2017_3291_MOESM2_ESM.tif]

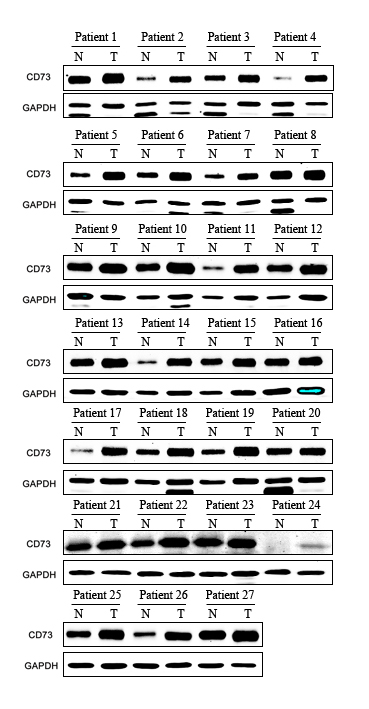

Supplement: Supplementary file 3 — The original results of western blot for the colorectal cancer tissues. (JPEG 153 kb) [file 12885_2017_3291_MOESM3_ESM.jpg]

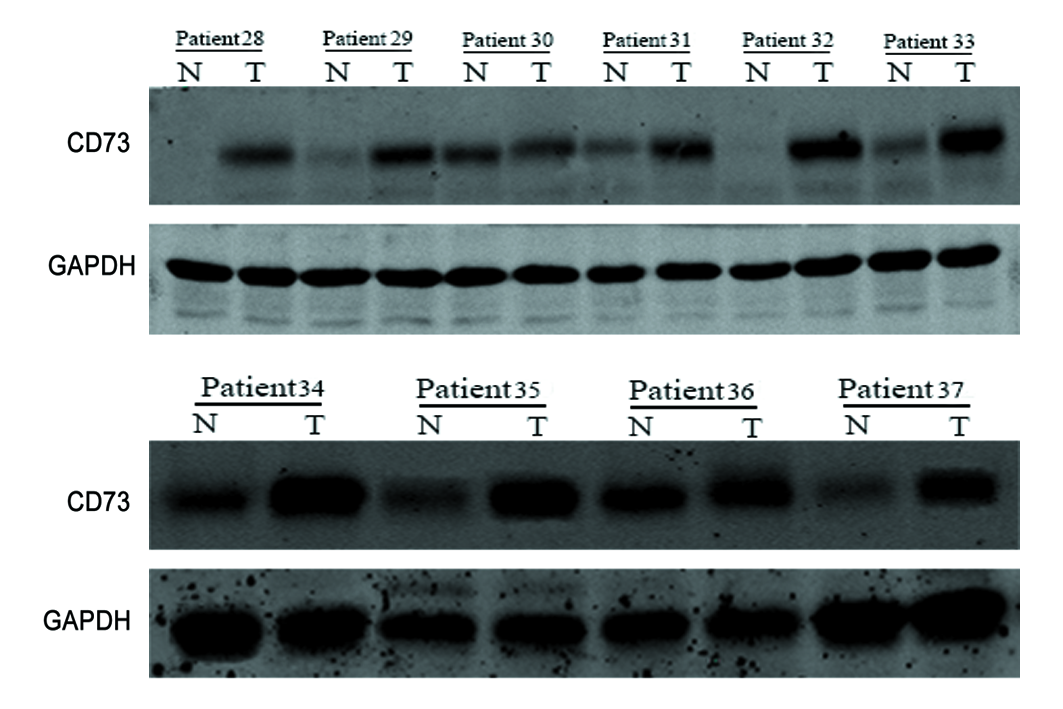

Supplement: Supplementary file 4 — The results of western blot for the new collected colorectal cancer tissues. (TIFF 2972 kb) [file 12885_2017_3291_MOESM4_ESM.tif]

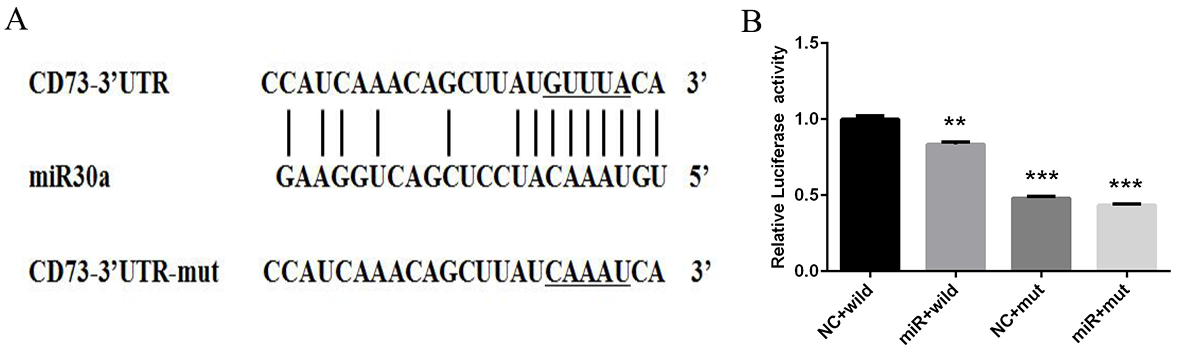

Supplement: Supplementary file 5 — A. Wild-type (WT) and mutant (Mut) of putative miR-30a targeting sequences in CD73 mRNA. Mutant sequences were shown in underline. B. The miR-30a target sequence from CD73 was cloned into the 3′-UTR of a luciferase reporter gene. Seed site mutagenesis was used to control for binding specificity. Luciferase activity was determined by Dual-Luciferase Reporter Assay System. Error bars represent mean ± SD from three independent experiments. *P < 0.05, **P < 0.01 compared with the NC group. (TIFF 568 kb) [file 12885_2017_3291_MOESM5_ESM.tif]
