# Supplementary material for: MicroRNA-30a regulates cell proliferation and tumor growth of colorectal cancer by targeting CD73
Source: BMC Cancer. 2017 May 2;17:305. doi: 10.1186/s12885-017-3291-8 (PMC5414330; doi:10.1186/s12885-017-3291-8)
Supplement: Supplementary file 6 — The scan of informed consent for preservation of the tissue specimens in Chinese. (PDF 816 kb) [file 12885_2017_3291_MOESM6_ESM.pdf]

中山大學附属第六医院
广东省胃肠肛门医院
THE SIXTH AFFILIATED HOSPITAL OF SUN YAT-SEN UNIVERSITY
GUANGDONG GASTROINTESTINAL HOSPITAL

关于保存组织库标本的知情同意书

患者姓名____________ 住院号_________ 性别_____ 年龄_____ 科室_______

临床诊断 ____

一、谈话内容：

组织标本是开展科学研究的重要资源，对人类认识肿瘤的发病规律、探索有效的预防和治疗方法具有不可替代的作用。

在临床规范化治疗中，临床检查需要必要地采集您的外周血、尿、便或其他体液等，手术治疗需要切除手术标本。通常情况下，上述标本在满足治疗和诊断之后会予以废弃处理，现我们可能会使用少许标本进行医学研究，以探索对于疾病的新的诊断和治疗方法。

对您而言，最大的风险是隐私及健康信息的泄露，但我们将对您的有关信息进行保护，您提供的样本将用一个编号表示，与您的姓名和编号联系的基本项目将以电子版档案的形式保存在计算机中，只有授权人才可以看到您的相关信息，研究人员不会知道您的身份。

最后，我们尊重您的选择，不论您是否同意保存剩余的组织样本，都不会影响您的治疗。

衷心感谢您对该种疾病研究与治疗进展所做的宝贵贡献！

二、患方意见

对于以上医生所述的各项内容，患者/监护人/家属表示已知情并同意接受组织标本的保存。

患者签名：______________ ______年___月___日 患者手印（必要时）：_________

患者委托人签名：______________与患者的关系：_______ _____年____月____日

医师签名：______________ _______年____月____日

说明：1、表格内容必须逐项认真填写、字迹清晰，不得漏项；医务人员签名必须签全名。

2、“谈话内容”如需增补，医务人员在书写增补内容后必须交患方逐项签名确认。

3、特殊情况下患者不能书写本人姓名是，如患者为文盲等，可指导其在“患者手印”处按手印。
